# Overexpression of the Novel Senescence Marker β-Galactosidase (GLB1) in Prostate Cancer Predicts Reduced PSA Recurrence

**DOI:** 10.1371/journal.pone.0124366

**Published:** 2015-04-15

**Authors:** Jennifer Wagner, Nathan Damaschke, Bing Yang, Matthew Truong, Chad Guenther, Johnathon McCormick, Wei Huang, David Jarrard

**Affiliations:** 1 Department of Urology, University of Wisconsin School of Medicine and Public Health, Madison, Wisconsin, United States of America; 2 Department of Pathology and Laboratory Medicine, University of Wisconsin School of Medicine and Public Health, Madison, Wisconsin, United States of America; University of Kentucky College of Medicine, UNITED STATES

## Abstract

**Purpose:**

Senescence is a terminal growth arrest that functions as a tumor suppressor in aging and precancerous cells and is a response to selected anticancer compounds. Lysosomal-β-galactosidase (GLB1) hydrolyzes β-galactose from glycoconjugates and is the origin of senescence-associated β-gal activity (SA-β-gal). Using a new GLB1 antibody, senescence biology was investigated in prostate cancer (PCa) tissues.

**Experimental Design:**

*In vitro* characterization of GLB1 was determined in primary prostate epithelial cell cultures passaged to replicative senescence and in therapy-induced senescence in PCa lines using chemotherapeutic agents. FFPE tissue microarrays were subjected to immunofluorescent staining for GLB1, Ki67 and HP1γ and automated quantitative imaging initially using AQUA in exploratory samples and Vectra in a validation series.

**Results:**

GLB1 expression accumulates in replicative and induced senescence and correlates with senescent morphology and *P16 (CDKN2)* expression. In tissue arrays, quantitative imaging detects increased GLB1 expression in high-grade prostatic intraepithelial neoplasia (HGPIN), known to contain senescent cells, and cancer compared to benign prostate tissues (p<0.01) and senescent cells contain low Ki67 and elevated HP1γ. Within primary tumors, elevated GLB1 associates with lower T stage (p=0.01), localized versus metastatic disease (p=0.0003) and improved PSA-free survival (p=0.03). Increased GLB1 stratifies better PSA-free survival in intermediate grade PCa (0.01). Tissues that elaborate higher GLB1 display increased uniformity of expression.

**Conclusion:**

Increased GLB1 is a valuable marker in formalin-fixed paraffin-embedded (FFPE) tissues for the senescence-like phenotype and associates with improved cancer outcomes. This protein addresses a lack of senescence markers and should be applicable to study the biologic role of senescence in other cancers.

## Introduction

PCa is the second leading cause of cancer-related death for men in the United States. However, the majority of men with this disease will die from other causes. Predicting the clinical behavior of PCa primarily relies on Gleason Score and other clinicopathologic factors [1]. However, these features incompletely describe the natural history of the disease. Although most markers have focused on the identification of more aggressive cancer behavior, few markers are overexpressed that signify a more indolent tumor behavior.

Senescence is a terminal growth arrest originally described in aging cells. Senescence results from telomere uncapping due to replicative exhaustion, mitochondrial deterioration, oxidative stress, severe or irreparable DNA damage or selected oncogene expression [2]. As such, it represents an important tumor suppressor mechanism to prevent cancer in normal cells. More recently it has been described in a subset of cancer cells after selected types of chemotherapy or radiation and identifies populations of growth-arrested cells [3]. This phenotype has been termed therapy-induced senescence (TIS) [4]. Indicative of a suppressive role, senescent cells have been demonstrated in lung adenomas, but not in associated lung cancers [5]. In the prostate, senescence markers are found in high grade prostatic intraepithelial neoplasia (HGPIN), a benign lesion associated with the presence of cancer [6]. These data suggest that the presence of senescence has the potential to indicate a more benign clinical course in tumors.

Specific markers of senescence have been lacking, especially those that can be employed in paraffin embedded, formalin-fixed tissues. GLB1 (lysosomal-β-galactosidase) is a lysosomal enzyme that hydrolyzes the terminal β-galactose from ganglioside substrates and other glycoconjugates [7]. The *GLB1* gene was found to be the source of senescence associated-β-gal activity (SA-β-gal) [8], and expression correlates with SA-β-gal activity both *in vitro and in vivo* [9]. Staining for SA-β-gal is the predominant method to identify senescent cells, but requires fresh or frozen tissue to assess enzymatic activity [10]. Other markers associated with, but not specific for senescence include a low proliferative activity, decreased p27 [6,11], as well as alterations in nuclear matrix genes including increased heterochromatin protein 1 gamma (HP1γ) [12].

In the current study, the specificity of a GLB1 antibody directed against the lysosomal portion of SA-β-gal was confirmed in cellular models of replicative senescence and of TIS. The expression of GLB1 was then examined in HGPIN, cancerous, and benign prostate tissues. Vectra, an advanced multispectral imaging system, was utilized to quantitatively measure multiple proteins simultaneously to evaluate expression within each cell compartment (epithelial vs. stromal) and within each cell (nuclear vs. cytoplasm) [13]. Indicative of its tumor suppressive nature, GLB1 was found to be highest in HGPIN, known to contain senescent cells [6] compared to benign and cancer tissues. Notably, higher GLB1 expression within prostate tumors is associated with more favorable clinicopathologic features and reduced PSA failure after surgery. These data demonstrate that GLB1 is a novel marker for more indolent PCa and that its accumulation suggests the increased presence of senescent cells in these tumors.

## Materials and Methods

### Cell Culture

Human prostate epithelial cells (HPEC) were cultured from fresh prostate tissues (confirmed to be free of cancer) obtained from radical prostatectomy specimens (ages 45–60) as previously described [14]. All tissues were obtained with written consent from patients; all protocols were reviewed and approved by the University of Wisconsin Institutional Review Board (IRB). The cells were maintained on collagen-coated plates in Ham's F-12 supplemented medium containing 1% fetal bovine serum. Matched proliferating cells (passage 1) were harvested and compared to senescent cells (passage 5 and above) containing >50% senescent cells by morphology and SA-β-gal activity [15]. As a model of TIS, Du145 and LNCaP cells were plated at a density of 16,000 and 32,000 cells, respectively, per well (6-well plate) and exposed to medium containing 250nM Aziridinylbenzoquinone (AZQ) for 72 hours. AZQ has previously been demonstrated to be a potent senescence inducer [11]. The cells were washed in phosphate-buffered saline (PBS) and cultured in drug-free medium, and then cell lysates were collected at 5 and 7 days after the drug treatment started.

### Western blot analysis

Cells were collected and solubilized in lysis buffer and transferred to nitrocellulose membranes. Membranes were blocked and probed with antibodies to GLB1 (#ab55176; AbCam, Cambridge, MA) in a 1:1000 dilution, p16 and p27 (Santa Cruz, Dallas, TX) in a dilution of 1:500, or α-tubulin (Sigma, St. Louis, MO) in a 1:2000 dilution, followed by HRP-conjugated anti-mouse or anti-rabbit (Thermo/ Pierce, Rockford, IL) in 1:5000. Band intensities were measured using ImageJ (http://rsbweb.nih.gov/ij/), background intensities were subtracted and normalized to α-tubulin intensities and expressed in relative units as a function of control band intensity. In each case, the immunoblot presented is representative of at least three experiments.

### Immunocytochemistry and Flow cytometry

Prostate cancer cells were fixed with 3.7% formaldehyde and permeablized with 0.3% Triton-100 after treatment with senescence inducing drugs. Blocking unspecific binding sites with blocking buffer for 1 hour at room temperature, the cells were then stained with mouse anti-human GLB1 antibody (AbCam, Cambridge, MA) in 1:200 dilution overnight at 4°C, labeled with anti-mouse Alexa 647 (Invitrogen, Grand Island, NY) in 1:1000 dilution and then detected with EVOS FL Auto Imaging System. To determine the relative cell size, granularity or internal complexity, prostate cancer cells were collected after treatment and live cells applied to MACSQuant Analyzer (Miltenyi Biotec Inc. San Diego, CA). The cell complexity in different groups was analyzed by side scatter (SSC).

### Tissue Microarray (TMA)

Formalin fixed paraffin embedded (FFPE) patient tissues tissue arrays were obtained from the University of Wisconsin Department of Pathology under University of Wisconsin Health Sciences IRB Office approval. All participants provided their written informed consent to participate in this study. The UW Institutional Review Board reviewed and approved all protocols used in this study. An exploratory array with no clinical follow-up included 41 localized PCa, 41 benign peripheral prostate tissues, 24 benign prostatic hyperplasia (BPH), and 18 HGPIN samples in duplicate cores (total 336 cores) as previously described [16]. A validation array in a separate population consisted of 292 cores in duplicate using tissues from 65 primary PCa specimens of which 38 were localized (pT2) and 27 higher stage (pT3 and 4). There were 25 HGPIN and 48 benign prostate tissues on the arrays. Characteristics and follow-up of this population are provided in Table 1.

**Table 1:** Clinicopathologic Characteristics of Validation Cohort.

Variable	Average	Number (%)
**Age**	58.9	
**PSA**	10.24	
**Tumor Volume (cc)**	0.19	
Gleason score*		
6		18 (32.7%)
7		30 (54.5%)
8		3 (5.5%)
9		4 (7.3%)
**Stage**		
II		35 (62.5%)
III		11 (19.6%)
IV		10 (17.9%)
**Metastatic**		
No		46 (82.1%)
Yes		10 (17.9%)
**Extraprostatic Extension**		
No		39 (69.6%)
Yes		17 (30.4%)
**SV Involvement**		
No		44 (80%)
Yes		11 (20%)
Biochemical Recurrence**		
No		31 (70.5%)
Yes		13 (29.5%)

* One patient missing clinical data

**44 Patients with complete biochemical recurrence follow-up

### AQUA and Vectra Immunostaining and Analyses

The TMA sections were taken through routine deparaffinization and rehydration, HIER treatment with citrate for 20 min, and staining as previously described [16]. For AQUA immunostaining, a mixture of primary antibodies (monoclonal mouse anticytokeratin, AE1/AE3, Dako, Carpinteria, CA, 1:200 and GLB1 #ab55176; AbCam, Cambridge, MA, 1:100) and secondary antibodies were applied to the TMA as described [16]. Prostate epithelium was distinguished from stroma with the cytokeratin antibody and an epithelial binary mask was created after pixel-based locale assignment for compartmentalization of expression (PLACE) algorithm where the stroma was removed. The AQUA score was determined as described.

Immunostaining for Vectra included GLB1 at a 1:100 concentration, E-cadherin antibodies (Cell Signaling Technology, Beverly, MA) at 1:200 were used to define the epithelial compartment for tissue segmentation. Antibodies for P27 (BD Biosciences, San Jose, CA) and HP1γ (EMD Millipore, Bilerica, MA) were used at 1:100 and 1:50 dilution, respectively. Detailed protocols are available on request. For automated image acquisition and analysis, the stained slides were loaded onto the slide scanner and per-cell GLB1 target signals were quantitated for individual cores using the Vectra imaging system according to manufacturer’s protocols (Caliper Life Sciences, Hopkinton, MA). InForm1.2 Software was used to segment tissue compartments (epithelium vs. stroma) and subcellular compartments (nucleus vs. cytoplasm). Both per cell and per core analyses were performed.

### Statistical Analysis

Nuclear/cytoplasmic and epithelial/stromal expression was statistically compared using a Students t-test. GLB1 protein expression was compared against clinicopathologic features (Gleason score, primary tumor (pT) stage, PSA, tumor volume, seminal vesicle (SV) involvement, margins, extracapsular extension, and evidence of metastasis) using a t-test. For this analysis, multiple PCa cores obtained from the same patient were first averaged. Linear regression was used to compare GLB1 expression to Ki67 expression in a given sample.

Heterogeneity was assessed by averaging cell expression levels within each core and then averaging all cores in each type of prostate tissue. Skewness, kurtosis, and coefficient of variation were calculated. Mann-Whitney U was used to calculate significance.

To determine percent of positively staining GLB1 cells, all cell expression levels were averaged and the mean was the cut-off point for a “positive” GLB1 stain or “negative.” Within each core, the percent of “positive” cells was determined. In a tissue type (HGPIN, cancer, and benign) this percentage value from each core was averaged to determine the overall percent positively staining cells in that tissue.

To analyze recurrence, the median value of all cores of benign and cancer tissues was used as the cut-off point for low versus high GLB1 staining. PCa patients were stratified based on high or low GLB1 levels. A Kaplan-Meier survival curve was plotted to assess survival times free of PSA recurrence, and a log rank (Mantel-Cox) analysis was done to determine significance. A p-value of <0.05 was considered statistically significant. Statistical analysis was performed using SPSS Version 20.0 (IBM).

## Results

### GLB1 expression is increased in HGPIN and primary PCa

Tissue arrays were generated and analyzed that included samples of benign peripheral prostate tissue, HGPIN, and PCa. Analysis of immunohistochemical staining for GLB1 was performed on an initial tissue array using AQUA (automated quantitative analysis), an automated scoring system for assessing biomarker expression within epithelial compartments [17]. Subsequent analysis of the validation array was performed using Vectra, a newer generation automated imaging and quantification approach that provides multichannel objective analysis of expression data.

**Fig 1:**
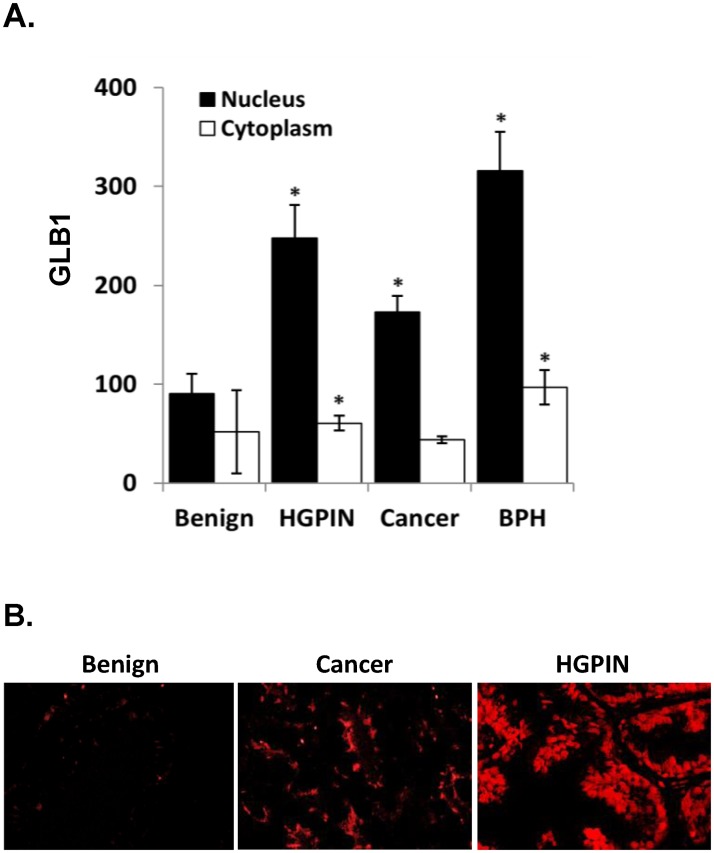
GLB1 increases expression in HGPIN and PCa. **(A)** Gene expression was measured using the AQUA platform as described [20] in an initial multitissue prostate array. Epithelial GLB1 expression is significantly increased in HGPIN, primary prostate cancer and BPH compared to benign in both nuclear and cytoplasmic compartments (All p<0.001). For all tissue types, expression was 2–3 times greater in the nucleus than the cytoplasm (p<0.0001). **(B)** Tissue microarrays immunofluorescently stained Glb1 (red) primarily in the epithelium.

Imaging and quantitative analysis of the initial array demonstrated significantly higher GLB1 expression in primary PCa and HGPIN (Fig 1A and 1B). HGPIN has previously been found to express increased SA-β-galactosidase activity and other markers [6, 18]. Benign prostatic hyperplasia (BPH) also increased GLB1 expression compared to benign peripheral prostate tissues consistent with increased SA-β-gal activity seen previously in these tissues [19].

### GLB1 expression is increased in replicative and induced senescent cell cultures

**Fig 2:**
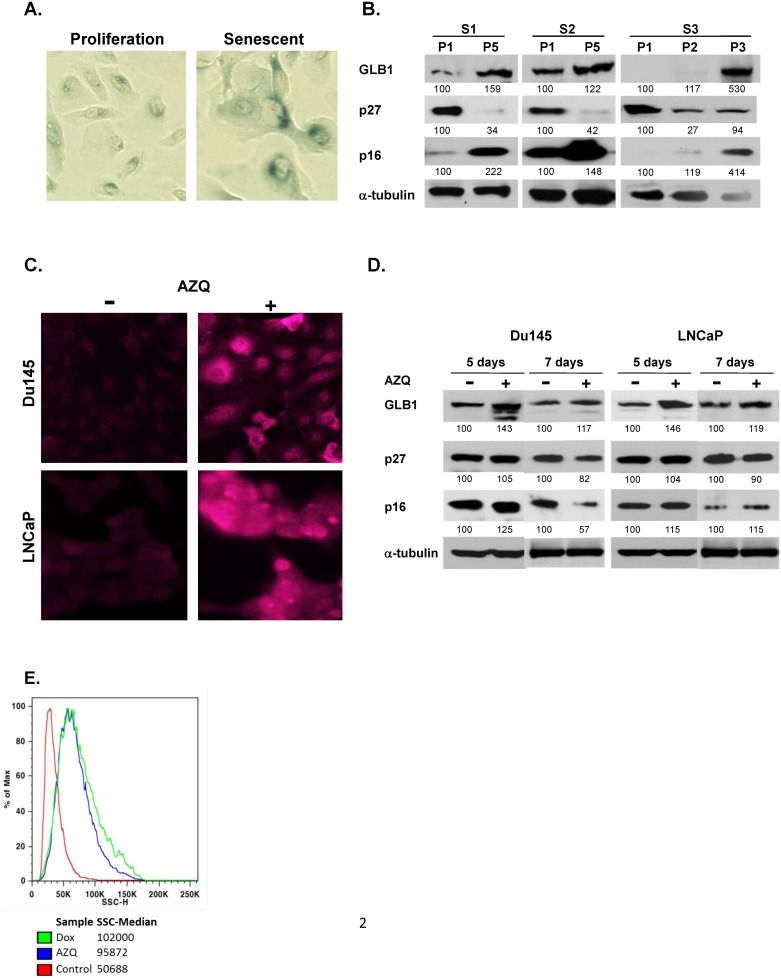
GLB1 is increased in replicative and therapy-induced senescent (TIS) cell culture models. **(A)** Human prostate epithelial cells were cultured to senescence by repeated cell passaging demonstrate an enlarged, more complex senescent cell morphology and increased β–gal staining in passage 5 (P5) than in proliferating (P1) cultures. **(B)** Western blot comparing proliferating HPECs (P1) versus later passages (P5) for separate cultures (S1, S2, S3) are shown. GLB1 and p16 expression is increased in senescence. Cultures contained >50% senescent cells by SA-βgal staining. Images were normalized to α-tubulin. **(C)** GLB1 mmunofluorescence increases in cancer cells displaying TIS. Du145 and LNCaP cells were treated with 250nM AZQ or DMSO (control) for 3 days, kept in drug-free media for an additional two days, then stained with mouse anti-human GLB1 antibody and detected with anti-mouse-Alexa 647. **(D)** Western blot analysis of GLB1 expression at 5 or 7 days after AZQ treatment performed in DU145 and LNCaP PCa lines. Cells were compared against a DMSO-treated control group (Con) and normalized to α-tubulin expression levels. **(E)** Cell granularity and complexity analysis of senescence by flow cytometry. Du145 cells were treated with AZQ or Doxorubicin for 3 days, kept in drug-free media for two days then collected and applied to MACSQuant flow cytometer immediately. Side scatter (SSC), which increases in senescent cells, increased in senescent AZQ and Doxorubicin treated cells compared to control.

The senescent phenotype is induced in culture in cancer cells using specific drugs, or with repeated cell passage of human prostate epithelial cells (HPECs) [15]. To assess the specificity of the GLB1 antibody to detect senescence, a series of HPEC cultures were analyzed after prolonged passaging. HPEC sample sets had greater than 50% senescent cells in later passages detected by SA-β-gal activity staining and morphology (Fig 2A). The senescent morphology includes cellular enlargement, increased cellular complexity and occasional multinucleation. Western blot in later passage HPECs displays increased GLB1 expression compared to proliferating primary cells (Fig 2B). P16, a marker of terminal senescence in HPECs [21], also demonstrates increasing expression that correlated with increased GLB1. P27 expression initially increases in early senescence, then decreases in terminal senescence as previously noted [11, 22]. Serum-starved arrested (quiescent) cultures show no GLB1 expression alterations or senescent morphologic changes as previously described [15].

To generate therapy-induced senescent (TIS) cancer cells, PCa cells were treated with Diazequone (AZQ) a quinone (250nM) and robust inducer of senescence in cancer cells as previously described [11]. After 5 days or 7 days, cultures of Du145 and LNCaP show cumulative increases in GLB1 expression by immunofluorescent staining (Fig 2C) and western blot (Fig 2D) compared to controls. Fluorescent labeling indicates that senescent cells express increased GLB1 in both the cytoplasm and nucleus. P16 demonstrates some accumulation in Du145 and LNCaP at 5d (Fig 2D), although some overgrowth of proliferating cells occurs in Du145 at 7d attenuating P16 protein at that timepoint. As previously demonstrated, p27 levels decreases in terminal senescence at day 7 [11]. Side-scatter (SSC), a phenotypic measure of the increased cellular size and complexity was determined using Flow Cytometry [9]. Both AZQ and low dose Doxorubicin, a known senescence-inducing agent, increases SSC at Day 5 when compared to controls (Fig 2E). These studies demonstrate the association of GLB1 with phenotypic and molecular markers of senescence *in vitro*.

### A subset of primary PCa samples express increased GLB1

**Fig 3:**
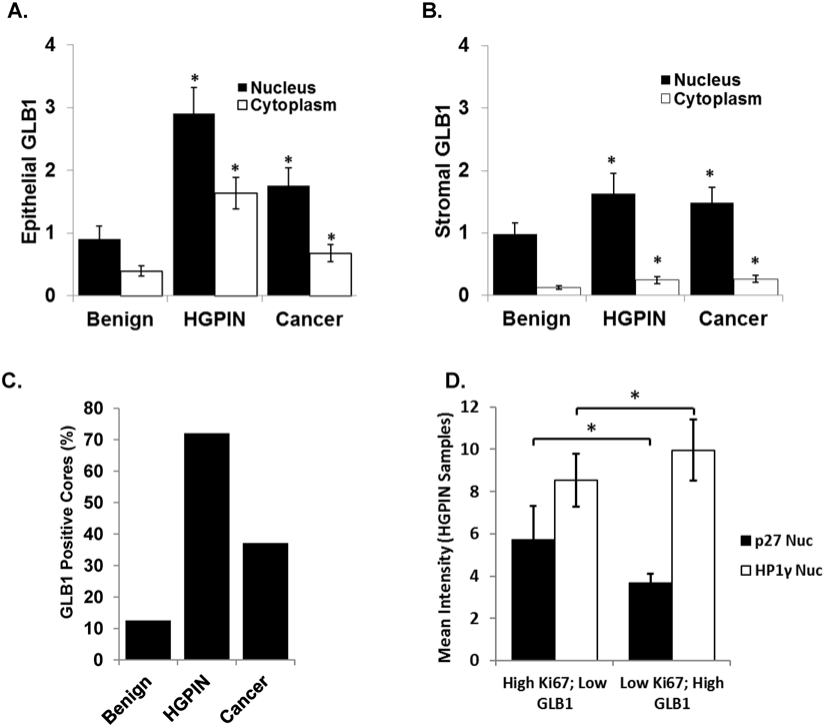
GLB1 increases expression in HGPIN and a subset of PCa and correlates with decreased proliferation and increased expression of HP1γ. Gene expression was measured using the Vectra platform in a larger tissue array. In all tissue types, nuclei stained more strongly for GLB1 than the cytoplasm (p<0.0001). Data are mean +/- 95% confidence intervals. * p<0.001. **(A)** Epithelial GLB1 expression is significantly increased in HGPIN and cancer compared to benign prostate in both nuclear and cytoplasmic compartments (All p<0.0001). **(B)** Stromal GLB1 expression levels in HGPIN and cancer exhibited similar intensities of staining (p<0.001 all). **(C)** The mean GLB1 expression was calculated for all samples and the number of cores that overexpressed GLB1 was determined for each pathologic entity. Primary cancer samples demonstrated increased expression in a subset of samples (nuclear expression shown). **(D)** The microarrays were immunostained for the proliferation protein Ki67, the heterochromatin protein HP1γ increased in senescence, and p27, a marker decreased in terminal senescence. Separating HGPIN cores into high and low Ki67 and GLB1 staining, the population of high GLB1-low Ki67 (right), representing the senescent population, expressed low p27 and high HP1γ as expected for senescent cells. This was significantly different from the expression of p27 and HP1γ in the low-GLB1-high Ki67 population (left), confirming the association of GLB1 with other senescent markers *in vivo*. Other subpopulations (high GLB1-high Ki67 and low GLB1-low Ki67) did not demonstrate significance and were omitted for clarity.

Additional tissue arrays from a patient cohort were analyzed that included pathological and clinical data as detailed in Table 1. Subsequent analysis of this validation array was performed using Vectra, a newer generation automated imaging and quantification approach that provides more defined tissue segmentation and multichannel objective analysis of multiple markers in addition to GLB1. Similar to the AQUA imaged array, increases in HGPIN and PCa compared to benign were observed in both the nuclear and cytoplasmic subsets (Fig 3A). In the epithelial compartment, nuclear expression was 2–3 fold greater than cytoplasmic in all tissues (p<0.0001). Stromal GLB1 expression levels were decreased compared to epithelial levels for all tissues analyzed (Fig 3B). Increased levels of stromal GLB1 were exhibited by HGPIN and cancer stroma (p<0.001 all), but the multifold increase in HGPIN epithelium was not observed. No correlation between GLB1 and age was seen in these normal or tumor samples (data not shown).

To calculate the number of samples that demonstrate altered GLB1 expression, the mean expression for all epithelial cells in all cores, benign and malignant, was calculated and then utilized as a cut-off point for determining positive or negative staining. Using this approach determined that 37% of primary cancer specimens, 72% of HGPIN samples and 13% of benign tissues had increased expression (Fig 3C). An alternate way of examining this overexpression is to evaluate the number of cells for each diagnosis that exceeded this value. On average, 51% of HGPIN cells (p<0.001) and 29% of cancer cells (p<0.001) stained positive compared to only 16% of benign specimens. These data indicate that GLB1 expression increases in a subset of primary cancer specimens.

### Increased GLB1 correlates with decreased proliferation and increased expression of the senescent marker HP1γ

To further define GLB1 significance in clinical samples, the arrays were immunostained for the proliferation marker Ki67, HP1γ whose nuclear expression increases in senescent cells [12], and p27, a cyclin-dependent kinase inhibitor that decreases in terminal senescence [11]. Analysis of benign and HGPIN tissue sets were performed since HP1γ alters expression in cancer [23]. Separating HGPIN cores into high and low Ki67 and GLB1 staining, the population of high GLB1-low Ki67 also expressed low p27 and high HP1γ as expected for senescent cells (Fig 3D). The staining in this senescent population significantly differed from the expression of p27 and HP1γ in the low GLB1-high Ki67 population. This pattern of low p27 and high HP1γ was also seen in benign prostate tissues with high GLB1-low Ki67 (S1 Fig). These data confirms an association of GLB1 expressing cells with other markers found in senescence and decreased proliferation *in vivo*.

### Increased GLB1 expression identifies favorable clinicopathologic features in primary PCa and decreased PSA recurrence rates

Clinicopathologic features of primary PCa samples were then correlated with GLB1 expression (Table 2). These analyses were performed comparing the epithelial compartments. Cores from nonmetastatic primary PCa cores had higher expression than the cores from PCa that had metastasized (nuclear p = 0.003 and cytoplasmic p = 0.01) (Table 2). An increase in nuclear GLB1 expression was also found in tumor specimens of lower stage (p = 0.008) (Table 2). Lower PSA levels, smaller tumor volume, and the absence of seminal vesicle (SV) involvement trended toward higher GLB1 expression but did not reach significance. Analysis of Gleason score revealed no significant difference between lower (GS6-7) and higher (GS8-9) grade cancers. In summary, increased GLB1 expression in primary PCa cores was associated with more favorable clinicopathologic features.

**Table 2:** Association of GLB1 with Clinicopathologic Characteristics in Primary PCa Samples.

Variable	Nucleus		Cytoplasm	
	Mean Intensity (SD)	p-value	Mean Intensity (SD)	p-value
**Gleason Score**				
6–7	1.84 (1.48)		0.74 (0.73)	
8–9	1.73 (1.28)	0.771	0.61 (0.48)	0.418
**Stage**				
II	1.94 (1.45)		0.75 (0.74)	
III	2.11 (1.47)	0.7407	0.81 (0.52)	0.7335
IV	0.97 (0.82)	**0.0075**	0.37 (0.36)	**0.0249**
**Presence of Metastases**				
No	1.98 (1.44)		0.76 (0.69)	
Yes	0.97 (0.82)	**0.0036**	0.37 (0.36)	**0.0108**
**Age**				
45–54	2.28 (1.60)		0.98 (0.92)	
55–64	1.43 (1.12)	0.0795	0.48 (0.42)	0.0637
65–74	2.05 (1.57)	0.6961	0.77 (0.60)	0.4789
**Extraprostatic Extension**				
No	1.85 (1.4)		0.72 (0.69)	
Yes	1.66 (1.36)	0.6504	0.63 (0.51)	0.5897
**SV Involvement**				
No	1.94 (1.35)		0.72 (0.66)	
Yes	1.26 (1.51)	0.2293	0.56 (0.63)	0.512
**PSA**				
≤5	2.26 (2.35)		1.09 (1.27)	
5<x<10	1.83 (1.37)	0.7746	0.65 (0.53)	0.5954
≥10	1.6 (0.7)	0.6683	0.61 (0.31)	0.5639
**Tumor Volume(cc)**				
<0.1	2.15 (1.65)		0.86 (0.81)	
0.1≤x<0.3	1.71 (1.19)	0.3846	0.63 (0.58)	0.3395
≥0.3	1.63 (1.34)	0.3504	0.59 (0.53)	0.3017

**Fig 4:**
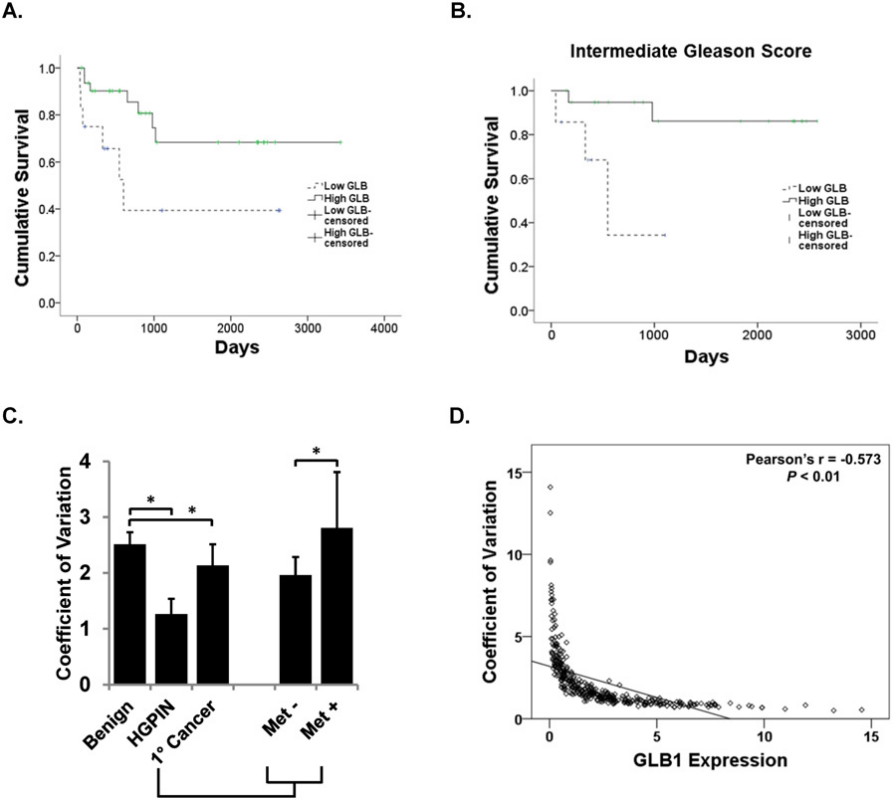
Increased GLB1 identifies patients at decreased risk of PCa recurrence. GLB1 expression on a per-core basis was measured using the Vectra platform in all tissues. Patients were stratified into high or low GLB1 levels based on median core expression. Kaplan-Meier survival curves are shown. **(A)** Increased GLB1 expression is associated with longer PSA-free recurrence in PCa patients. Stratified by low (n = 12) and high GLB1(n = 32) expression, cumulative PSA-free survival significantly increases in patients who had high GLB1 levels in a log rank analysis (p = 0.025). **(B)** High (n = 20) and low (n = 7) GLB1 levels predict PSA-free survival times (629 vs. 2314 days, respectively; p = 0.013) in intermediate Gleason Score (GS 6–7) patients. **(C)**. Per cell GLB1 expression is more homogenous in lower grade PCa tissues which express increased GLB1. GLB1 expression on a per-cell basis was then measured using the Vectra platform in all tissues. HGPIN had the least heterogeneity (p<0.001). Primary cancer tissue overall has significantly less heterogeneity than benign (p<0.001). Primary cancer cells with associated metastatic disease (Met+) had lower levels compared to localized tumors without the presence of metastatic (Met-) disease (p = 0.001) **(D)**. Mean nuclear GLB1 expression was inversely correlated with the coefficient of variation (representing heterogeneity) of the cores (Pearson's r = -0.573; p<0.01; N = 265). Tissues that display the highest levels of GLB1 staining have the most homogenous staining.

Recurrence-free survival was subsequently analyzed in PCa patients, and higher GLB1 expression associated with longer PSA recurrence-free survival (Fig 4A). Patients were stratified into high or low GLB1 levels based on the all core median expression. Cumulative PSA-free survival was significantly increased in patients with high GLB1 levels in a log rank analysis (p = 0.025). Mean survival was 1233 days with low GLB1 versus 2546 days with high GLB1. Patients with high Gleason Scores did not have significantly different PSA free survival based on GLB1 levels (p = 0.623). However, when intermediate GS 6–7 were analyzed separately, low and high GLB1 levels significantly differentiated PSA-free survival times (629 vs. 2314 days, respectively; p = 0.013) (Fig 4B). These results confirm that more favorable clinicopathologic outcomes are seen in samples expressing higher GLB1 levels.

### GLB1 heterogeneity decreases in cancer compared to benign tissues

Vectra allows a comparison of GLB1 expression in individual cells within cancer, HGPIN, or benign epithelium. There has been interest in the biological significance of expression variation since the few proteins analyzed to date in cancer demonstrate increased heterogeneity [24]. An analysis of heterogeneity was performed across all prostate tissue cores using per cell data (Fig 4C). Our first analysis found that the heterogeneity of GLB1 staining decreases in HGPIN (p = 0.001) suggesting that as cells became arrested in the senescent state, GLB1 becomes more homogeneous. Cancer also displayed less heterogeneity of GLB1 when compared to benign prostate tissue. Primary PCa cores associated with the presence of metastatic disease had increased heterogeneity when compared to other primary samples. There was no significant difference across Gleason Scores in GLB1 heterogeneity (data not shown).

A correlation analysis was performed further compare core heterogeneity and GLB1 expression in the nuclear epithelium. Across all tissues, a negative correlation was found (Pearson's r = -0.573; Sig 0.00; N = 265) indicating that when GLB1 protein expression is highly expressed, heterogeneity is lower (Fig 4D). This correlation was also significant when benign, HGPIN, and cancer were analyzed separately (-0.719, -0.603, and-.567 respectively). This indicates that tissues displaying the highest levels of GLB1 staining also had the homogeneity of expression.

## Discussion

Senescence is an important tumor suppressor mechanism in human cells. Its study in human tumors has been limited by an absence of senescence markers that can be utilized *in vivo* especially in FFPE tissues. In the current paper, we demonstrate using an antibody targeted against the lysosomal protein GLB1 that its expression correlates with the senescence phenotype in several *in vitro* models. In human tissues, the highest GLB1 expression levels are found in HGPIN, a premalignant lesion in the prostate [25]. This is consistent with the previous finding of other senescence markers in HGPIN including increased p16 and SA-β-gal activity [6, 18]. Interestingly, GLB1 expression is more homogeneous in highly expressing samples. Only a subset of primary PCa expressed higher levels of GLB1, and these tumors had more favorable clinicopathologic characteristics including PSA-free survival after treatment. The biological impact of senescent-like cells within tumors, whether they play an inductive or inhibitory role, is an area of active debate. The current data supports the hypothesis that the presence of senescence cells, at least in primary cancer tissues, indicates more biologically favorable tumor behavior.

The protein levels of GLB1 detected by this antibody were first assessed in a well-described model of senescence induced after repeated population doublings in human prostate cells [26]. These mortal prostate cells demonstrate increased SA-β-gal activity, the typical “fried egg” senescence morphology and have been used to identify other senescence expression markers [15, 22]. An increase in GLB1 was found in this model and confirmed by also comparing p16 accumulation, a well-established senescence marker [21]. Proliferative cells have less GLB1, and this protein accumulates as the cell transitions to later stages of senescence. Senescence has also been identified after selected chemotherapy and has been termed Therapy-Induced Senescence (TIS) [3]. We demonstrate increased expression of GLB1 in a TIS model using AZQ, a potent senescence inducer [4]. These dual models confirm its applicability as a measure of senescence *in vivo*. We also correlated low proliferation-high GLB1 with several previously described markers of senescence in the tissues arrays. HP1γ elevation has been shown in several models to increase in senescence [27] and p27 decreases in terminal senescence [11].

It has been postulated that senescence in cancer tissues may predict tumor outcomes since it may indicate, as in premalignant samples, an increased opposition to malignant transformation and growth [3]. In colon cancer, tumor biopsies show senescent areas after 5-Fluorouracil and Leucovorin treatment demonstrate longer progression-free survival than patients without senescence [28]. This correlation, however, has not been demonstrated in primary, untreated cancers to date in part due to a lack of markers functional in FFPE samples. The Vectra automated analysis revealed PCa samples have increased levels of GLB1 compared to benign prostate. A novel observation is that increased GLB1 expression indicates more favorable clinicopathologic parameters including lower stage and localized disease. Extraprostatic extension and seminal vesicle involvement demonstrate lower GLB1 levels. Notably, PSA recurrence-free survival significantly increased in PCa patients that had high GLB1 levels. In intermediate grade cancers, which can pose a management dilemma whether active surveillance or treatment might be utilized, this marker should be of use with additional validation.

Tumors demonstrate an increased heterogeneity in cellular morphology, reflected in altered staining, but automated quantitative analysis of protein expression in cancer has not been extensively studied. For the majority of cell protein expression, heterogeneity increases with tumor formation indicating clonal diversity [29]. The per-cell expression of GLB1 was obtained using the Vectra system generating several million observations, and the coefficient of variation was calculated. The precancerous lesion HGPIN demonstrated the lowest heterogeneity (highly homogeneous) for GLB1 staining, and also had the highest expression of this protein (Figs 3A and 4C). We speculate these findings represent the rapid replication of the majority of HGPIN cells to the senescent block and their accumulation at this point. Alternatively, it may be reflective of a more clonal origin for HGPIN [30]. Localized cancer appears more similar to HGPIN, demonstrating less heterogeneity and increased numbers of GLB1 positive, senescent-like cells than primary tumors that have given rise to metastatic disease (Table 2, Fig 4C). A correlation analysis confirmed an inverse association between GLB1 expression and heterogeneity (Pearson's r = -0.573) (Fig 4D). This accumulation of cells in senescence as a homogenous population could provide a more defined target, compared to the typical heterogeneity tumors display, when considering the induction of senescence as cancer therapy. Recent studies have suggested senescent cells may provide a susceptible population in combination with drugs that alter metabolism in a synthetic lethal approach [31].

Genetic studies have demonstrated that SA-β-gal activity is encoded by the lysosomal-β-galactosidase gene and that levels of lysosomal-β-galactosidase protein increase during senescence [8]. SA-β-gal may not always be a specific marker of senescence per se, but rather a surrogate marker for increased lysosome number or activity and may be uncoupled from senescence in some cancer cell lines [8]. Increased GLB1 protein, especially in cancer cell lines, may therefore not always be specific for senescence. Interestingly, the majority of GLB1 protein was found in the nuclear component by fluorescent Vectra analysis rather than the cytoplasm (2–3 fold lower). On higher power imaging this staining represents perinuclear staining possibly associated with GLB1 processing in the endoplasmic reticulum. Further unpublished work using colorimetric rather than fluorescent staining finds higher levels of GLB1 in the cytoplasmic portion suggesting differences may be due to image processing.

The finding of increased senescence in low risk cancers suggests that the presence of senescent cells in tumors may be associated with more indolent behavior. This is consistent with other observations in premalignant tissues including melanotic naevi and mouse lung adenomas [5, 24]. However, there is a body of conflicting data mainly performed in cell culture that suggests that the senescence-associated secretory phenotype creates a microenvironment favorable to paracrine stimulation [32, 33]. Our observation of lower cancer recurrence rates fails to support a significant promoting role for senescent-like cells in primary cancer, however it may be the promoting phenotype may play a role in other circumstances. The identification of markers of indolent cancer is important given increasing clinical interest in active surveillance for lower risk PCa. The current data support the marker GLB1 as a measure of improved prognosis in primary PCa. It provides a marker in paraffin-embedded preserved tissue samples to identify clinical outcomes and will be widely applicable to studying the biology of senescence in other tumor systems.

## Supporting Information

S1 Fig Increased GLB1 correlates with decreased proliferation and increased expression of HP1γ in benign tissue. The microarrays were immunostained for the proliferation protein Ki67, the heterochromatin protein HP1γ increased in senescence, and p27, a marker decreased in terminal senescence. Separating benign cores into high and low Ki67 and GLB1 staining, we found that the population of high GLB1-low Ki67 (box 4), representing the senescent population, expressed low p27 and high HP1γ as expected for senescent cells. This was significantly different from the expression of p27 and HP1γ in the low-GLB1-high Ki67 population (box 1). These results are similar to those seen in HGPIN tissues. The number by * shows the same group compared to each other. The significant differences are *1 = p <0.001, *2 = p = 0.01, *3 = p = 0.015, *4 = p<0.001.
(TIF)
