# Supplementary figures and images for: Overexpression of the Novel Senescence Marker β-Galactosidase (GLB1) in Prostate Cancer Predicts Reduced PSA Recurrence
Source: PLoS One. 2015 Apr 15;10(4):e0124366. doi: 10.1371/journal.pone.0124366 (PMC4398352; doi:10.1371/journal.pone.0124366)

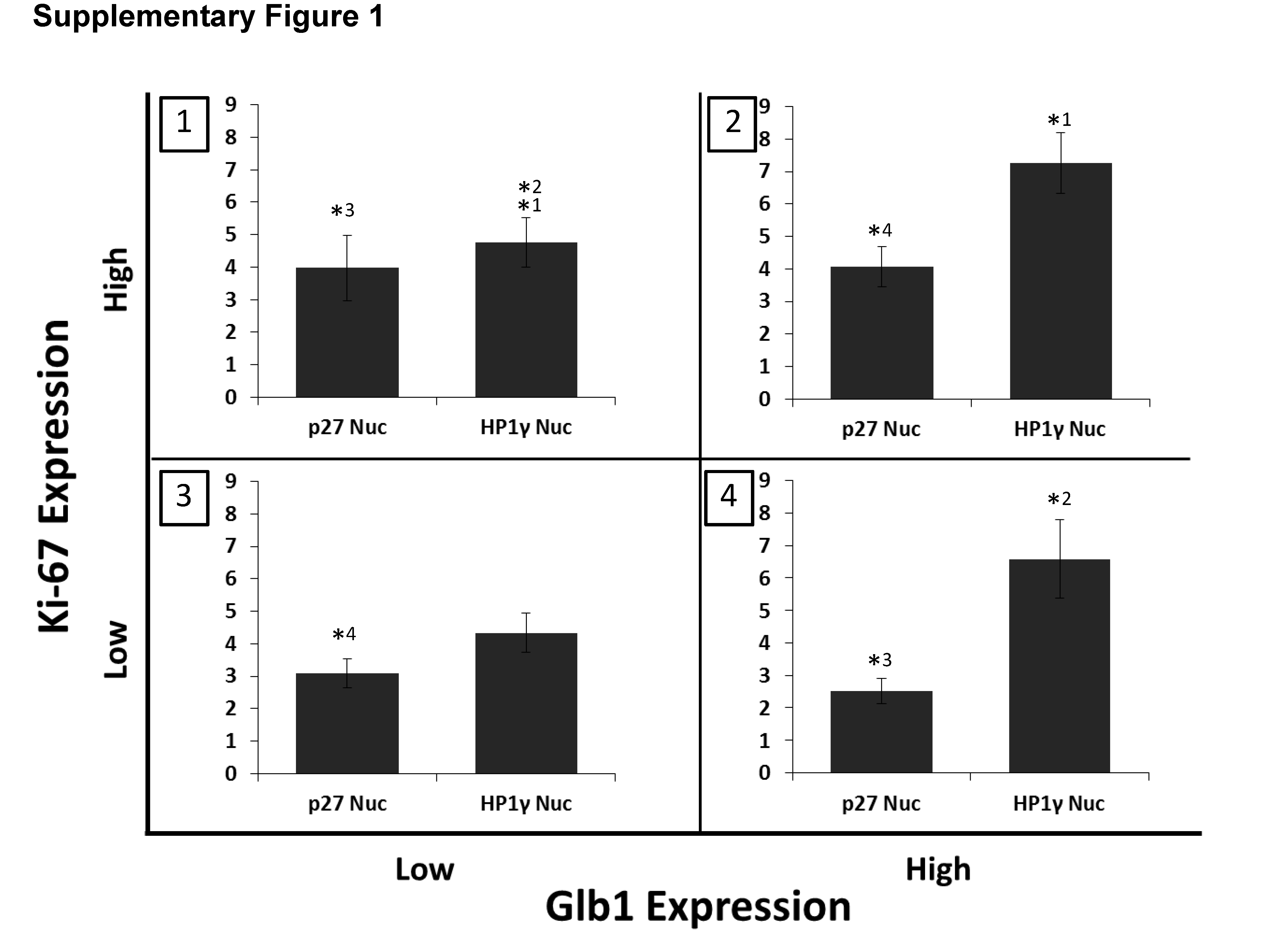

Supplement: S1 Fig — The microarrays were immunostained for the proliferation protein Ki67, the heterochromatin protein HP1γ increased in senescence, and p27, a marker decreased in terminal senescence. Separating benign cores into high and low Ki67 and GLB1 staining, we found that the population of high GLB1-low Ki67 (box 4), representing the senescent population, expressed low p27 and high HP1γ as expected for senescent cells. This was significantly different from the expression of p27 and HP1γ in the low-GLB1-high Ki67 population (box 1). These results are similar to those seen in HGPIN tissues. The number by * shows the same group compared to each other. The significant differences are *1 = p <0.001, *2 = p = 0.01, *3 = p = 0.015, *4 = p<0.001. (TIF) [file pone.0124366.s001.tif]
